# High Fecal Carriage of Multidrug Resistant Bacteria in the Community among Children in Northwestern Tanzania

**DOI:** 10.3390/pathogens11030379

**Published:** 2022-03-21

**Authors:** Delfina R. Msanga, Vitus Silago, Tulla Massoza, Benson R. Kidenya, Emmanuel Balandya, Mariam M. Mirambo, Bruno Sunguya, Blandina Theophil Mmbaga, Eligius Lyamuya, John Bartlet, Stephen E. Mshana

**Affiliations:** 1 Department of Pediatrics and Child Health, Weill Bugando School of Medicine, Catholic University of Health and Allied Sciences, Mwanza P.O. Box 1464, Tanzania; sylvestertullah@gmail.com; 2 Department of Microbiology and Immunology, Weill Bugando School of Medicine, Catholic University of Health and Allied Sciences, Mwanza P.O. Box 1464, Tanzania; vsilago.silago2@gmail.com (V.S.); mmmirambo@gmail.com (M.M.M.); stephen72mshana@gmail.com (S.E.M.); 3 Department of Biochemistry, Weill Bugando School of Medicine, Catholic University of Health and Allied sciences, Mwanza P.O. Box 1464, Tanzania; benkidenya@gmail.com; 4 Department of Physiology, School of Medicine, Muhimbili University of Health and Allied Sciences, Upanga West, Dar es Salaam P.O. Box 65001, Tanzania; ebalandya@yahoo.com; 5 Department of Community Health, School of Public Health and Social Sciences, Muhimbili University of Health and Allied Sciences, Upanga West, Dar es Salaam P.O. Box 65001, Tanzania; sunguya@gmail.com; 6 Kilimanjaro Clinical Research Institute, Kilimanjaro Christian Medical University College, Moshi P.O. Box 2236, Tanzania; blaymt@gmail.com; 7 Department of Microbiology and Immunology, Muhimbili University of Health and Allied Sciences, Upanga West, Dar es Salaam P.O. Box 65001, Tanzania; eligius_lyamuya@yahoo.com; 8 Duke Global Health Institute, Duke University Medical Center Durham, P.O. Box 3238, Durham, NC 27710, USA; bartl004@duke.edu

**Keywords:** children, MDR/ESBL colonization, human immunodeficiency virus

## Abstract

Colonization of multidrug resistant (MDR) bacteria is associated with subsequent invasive infections in children with comorbidities. This study aimed to determine the resistance profile and factors associated with MDR pathogen colonization among HIV−and HIV+ children below five years of age in Mwanza, Tanzania. A total of 399 (HIV− 255 and HIV+ 144) children were enrolled and investigated for the presence of MDR bacteria. The median [IQR] age of children was 19 (10–36) months. Out of 27 *Staphylococcus aureus* colonizing the nasal cavity, 16 (59.5%) were methicillin resistant while 132/278 (47.2%) of Enterobacteriaceae from rectal swabs were resistant to third generation cephalosporins, with 69.7% (92/132) exhibiting extended spectrum beta lactamase (ESBL) phenotypes. The proportion of resistance to gentamicin, amoxicillin/clavulanic acid and meropenem were significantly higher among HIV+ than HIV− children. A history of antibiotic use in the last month OR 2.62 [1.1, 6.9] (*p* = 0.04) and history of a relative admitted from the same household in the past three months OR 3.73 [1.1, 13.2] (*p* = 0.03) independently predicted ESBL rectal colonization. HIV+ children had significantly more fecal carriage of isolates resistant to uncommonly used antibiotics. There is a need to strengthen antimicrobial stewardship and Infection Prevention and Control (IPC) programs to prevent the emergence and spread of MDR pathogens in children.

## 1. Introduction

Antimicrobial resistance (AMR) is a global public health threat; it is estimated that it will kill about 10 million people annually by year 2050 [1]. AMR affects all countries, but the burden is disproportionately higher in low- and middle-income countries (LMIC) than in high-income countries (HIC) [2]. In many LMIC, a high burden of infectious diseases, poverty, weak governance and health systems, and low awareness remain major challenges in the fight against AMR [3,4].

Infection with AMR leads to serious illnesses, treatment failure, prolonged hospital stays and increased healthcare costs [5,6]. LMICs bear the consequences of AMR owing to the high cost and poor access to effective antibiotics to treat MDR infections [7]. MDR colonization is a risk factor for subsequent MDR infections [8,9,10] due to the fact that these MDR pathogens may serve as endogenous reservoirs for overt clinical infections. Overt subsequent clinical infections following colonization are more common in critically ill patients with comorbidities such as HIV, Diabetes Mellitus and Sickle Cell Disease [9,11].

HIV infection has been reported to be a risk factor for colonization with MDR pathogens [12]; this has partly been attributed to trimethoprim/sulfamethoxazole prophylaxis that is routinely given to prevent opportunistic infections [13,14]. The high prevalence of HIV and the emergence of MDR complicate efforts to control bacterial infections in many LMIC settings.

Evidence with regards to AMR is limited; however, it is known that HIV infected children below five years of age are particularly at risk of colonization with methicillin resistant *Staphylococcus aureus* (MRSA) and extended spectrum beta-lactamase producing Enterobacteriaceae (ESBL-PE) [15,16,17]. Therefore, this study aimed to determine the resistance profile and factors associated with MDR pathogen colonization among HIV− and HIV+ children below five years of age in Mwanza, Tanzania.

## 2. Results

### 2.1. Socio-Demographic and Clinical Characteristics of Enrolled Children

A total of 399 children below five years of age were enrolled with a median [interquartile range [IQR] age of 19 [10,11,12,13,14,15,16,17,18,19,20,21,22,23,24,25,26,27,28,29,30,31,32,33,34,35] months and 212 (53%) were males. The gender distribution was almost equal between two groups (*p* = 0.463). Antibiotic uses in the past one month (excluding SXT) was 89% in HIV− group and 90.3 in HIV+ group, *p* = 0.694 (Table 1). Among the enrolled HIV+ children, 143 (99.3%) were on ART.

### 2.2. Isolated Bacteria Pathogens from Nasal, Oral and Rectal Swabs

Of 399 enrolled children, 27 (6.8%) had nasopharyngeal colonization with *Staphylococcus aureus*. Of them, 16 (59.5%) were found to be MRSA. No significant difference of MRSA colonization was observed among HIV− children and HIV+ (13, 61.9% vs. 3, 50.0%; *p* = 0.662). The predominant pathogen colonizing the oral cavity was *K. pneumoniae* 11 (40.7%), followed by *E. coli* 5 (18.5%) and *S. pneumoniae* 4 (14.8%). Of 278 (69.7%) children screened for colonization of ESBL-PE, 92 (33.1%) were found to be colonized. The predominant isolates were *E. coli* 177 (63.7%) and *K. pneumoniae* complex 29 (10.4%) (Table 2).

### 2.3. Patterns of Resistance by Source of Bacteria

Gram negative bacteria from rectal isolates were significantly more resistant to ciprofloxacin, amoxicillin/clavulanic acid and ceftriaxone than Gram negative bacteria from oral cavity (48% vs. 16.7%, *p* = 0.007; 48.5% vs. 22.2%. *p* = 0.025; and 47.5% vs. 16.7% *p* = 0.009), respectively. Gram negative enteric pathogens from rectal isolates exhibited higher ESBL phenotype than those from the oral cavity (33.1% vs. 11.1% *p* = 0.039), (Table 3).

### 2.4. Comparison of Percentage Resistance of Gram-Negative Bacteria Colonizing Gastrointestinal Tract (GIT) of HIV Infected and Non-HIV Infected Children

Out of 278 isolates from GIT, isolates from HIV+ children had high resistance to gentamicin (28.2% vs. 8.3%, *p* < 0.001), amoxicillin/clavulanic acid (62.4% vs. 42.5%, *p* = 0.002) and meropenem (16.5% vs. 3.1%, *p* < 0.001) compared to those isolates from HIV− children. Although not statistically significant, HIV+ children were more colonized with ESBL-PE than HIV− children (42.4% vs. 30.6%, *p* = 0.056), (Table 4).

### 2.5. Multidrug Resistance Patterns among HIV and No HIV Infected Children below Five Years of Age

Among 278 GIT isolates, HIV+ children had significantly higher multidrug resistance to SXT/TET/CN (22.4% vs. 6.2% *p* = 0.000), CRO/CIP/CN (16.5% vs. 6.7% *p* = 0.012), TET/CN/CRO (17.7% vs. 5.2% *p* = 0.001), CN/TET/CIP (18.8% vs. 5.2% *p* = 0.0001), and SXT/TET/CN (22.4% vs. 6.2%, *p* < 0.001) (Supplementary File S1).

### 2.6. Factors Associated with ESBL Colonization among HIV− and HIV+ Children below Five Years of Age

On univariate logistic regression analysis, a history of antibiotic use in the last month (3.45, 95% CI: 1.39–8.58, *p* = 0.007) and a relative admitted from same household in the past three months (OR 4.8, 95% CI: 1.47–15.36, *p* = 0.009) were significantly associated with ESBL producing enteric bacteria GIT colonization in children below five years of age.

No significant difference was observed regarding ESBL colonization among HIV− and HIV+ children (25.0% vs. 23.14%); however, we found the proportion of ESBL-producing isolates to be higher in HIV+ children than in HIV− children, although this did not reach statistical significance (42.4% vs. 30.6%, *p* = 0.056). On multivariable logistic regression analysis, antibiotic use in the last month (OR 2.62, 95% CI: (1.1–6.9), *p* = 0.04) and history of a relative admitted from the same household in the past three months (OR 3.73, 95% CI (1.1, 13.2), *p* = 0.03) remained significant factors associated with ESBL producing enteric Gram-negative GIT colonization in children below five years of age (Table 5). The effect of trimethoprim/sulfamethoxazole (TSX) prophylaxis on colonization with EBSL could not be assessed as all HIV+ children were on TSX prophylaxis.

## 3. Discussion

This study reports the burden and associated factors of nasopharyngeal, oral, and rectal colonization of MDR pathogens in HIV− and HIV+ children below five years of age. About 4% of the enrolled children had nasopharyngeal colonization with MRSA, and 33.1% (92/278) were orally and GIT colonized by ESBL-PE, with no significant differences between HIV− and HIV+ children.

This study has shown a MRSA prevalence of 4%, similar with the values reported in China (3.9%) [18] and Taiwan (3.5–3.8%) [19], but lower than those reported in India (16.5–23.5) [19]. The low prevalence in the current study could be due to improvements in Infection Prevention and Control (IPC) procedures. It should be noted that the current study was conducted during the midst of the coronavirus disease 2019 (COVID-19) pandemic, where IPC, especially hand hygiene, was largely strengthened. In most developed countries such as Switzerland, the very low prevalence of MRSA colonization is largely related to routine screening, stringent IPC procedures and antimicrobial stewardship measures. The literature has documented that MRSA colonization occurs in individuals who have frequent exposure to healthcare settings, and in those with frequent antibiotic usage as well as those with immunodeficiencies [20,21].

In contrast to the low prevalence of MRSA colonization, the current study observed that almost two-thirds of children were colonized with potential ESBL-PE with a predominance of *E. coli* and *K. pneumoniae* complex. Similar findings regarding the colonization of ESBL producing *E. coli* and *K. pneumoniae* complex were reported in the same region among admitted neonates and street children [8,22], as well as other settings in LMIC [23,24,25,26,27] among non-HIV infected children. However, the prevalence was twice as high as that reported in HIC among non-HIV infected children [28], which could be attributed to poor hygiene, infrastructure and water sanitation, as documented previously in a meta-analysis [29]. Moreover, the misuse of antimicrobials is common in LMICs; it has been previously found to be associated with ESBL-PE carriage [2], as was confirmed in this study.

In this study we found that history of recent antibiotic use in the last month and a relative admitted living from same household in the last one month independently predicted ESBL producing enteric bacteria colonization. These findings are in line with studies done on admitted children in HIC, which documented that colonization was found to increase with a hospitalized family member in the three previous months [30,31]. Previous studies observed that children who had recent antibiotic usage had three times the odds of rectal colonization of ESBL-PE as opposed to those who had not been on antibiotics [24,32]. A family member with a history of hospital admission could carry and transmit a resistant pathogen to other family members due to poor hygiene and sanitation. One review documented that antibiotics commonly used in primary health care influence the composition of the gastrointestinal microbiota [33], increasing selection pressure on MDR pathogens. This process may be further complicated by poor IPC practices and hygiene which favor the spread of resistant bacteria in both the community and healthcare settings. Therefore, exposure to antibiotics and hospital admission increase the risk of becoming colonized with ESBL-GNB.

### Limitations

A correlation between colonization of ESBL and MRSA strains and subsequent occurrence of invasive infections was established in the current study. In addition, due to the fact that participants were children up to 5 years of age and most of information was retrieved from the parents, there is a possibility of recall bias, especially on the data regarding breastfeeding. We only collected data on overall duration of breastfeeding rather than duration of exclusive breastfeeding in order to mitigate this bias.

## 4. Materials and Methods

### 4.1. Study Design, Duration and Study Area

This cross-sectional study was conducted from April to December 2020 at Baylor Children’s Hospital and Nyamagana District hospital in Mwanza Tanzania. The study was designed to compare the burden and characteristics between HIV− and HIV+ children. Baylor Children’s Hospital is a patient-centered, pediatric HIV prevention and treatment clinic which serves the districts of Mwanza regions and other nearby regions. The clinic attends an average of 100 children per week. Nyamagana District Hospital runs a Reproductive and Child Health (RCH) clinic daily, attending approximately 30 children per day.

### 4.2. Study Population and Inclusion Criteria

All children aged six months to five years of age attending HIV outpatient clinic at Baylor Children’s Hospital and the Reproductive and Child Health Center at Nyamagana District Hospital were enrolled. The study included all children with no clinical signs and symptoms of systemic infection such as fever, chest wall indrawing, difficulty in breathing, crying during micturition, and diarrhea among other symptoms [34]. Children who had no documentation of HIV status on their RCH card and who failed to provide sufficient samples were excluded.

### 4.3. Sample Size Estimation, Sampling Technique

The study aimed at enrolling 125 children per group using a two independent proportions formula [35]. We used 16.9% as proportion 1 from the Tellevik study [24] and aimed to observe a 25% minimum difference; therefore, proportion 2 in the formula was assumed to be 42% with a beta level of 90% and an alpha level of 5%, giving a minimum sample size of 68 participants per group.

### 4.4. Data Collection and Sampling Procedures

A convenience sampling method was used to select the clinics based on proximity to a reference laboratory at Bugando Medical Centre. Children attending these clinics were enrolled serially until the sample size was reached. A data collection tool was customized in epicollect5 and used to collect socio-demographic characteristics (age, sex, socio-economic, household size etc.) and clinical data (duration on HIV treatment, history of antibiotics usage, vaccination history, vitals and anthropometric measurements and other comorbidities).

Anthropometric parameters which included height and weight were measured using a measuring tape and mechanical weight scale (SECA, Benson Avenue, New York, NY, USA) while the ages of the children were obtained from mothers/caregivers. In every enrolled child the Mid Upper Arm Circumference (MUAC) was measured and interpreted accordingly. Acute malnutrition is divided into severe acute malnutrition (SAM), being defined as a weight-for-height Z-score (WHZ) <−3 or a mid-upper arm circumference of <11.5 cm, or moderate acute malnutrition (MAM), with a WHZ between −2 and −3 Z-scores or a MUAC between 11.5 and 12.5 cm [36].

### 4.5. Sample Collection and Laboratory Procedures

#### 4.5.1. Samples Collection

Specimens (oral swab, nasal pharyngeal swab and stool/rectal swabs) were collected from all enrolled children by trained medical personnel. Sterile swabs (Mast Diagnostica GmbH, Reinfeld, Germany) in Amies transport media were used to collect a single time swab from every participant. Samples were then transported to the Microbiology Laboratory of the Catholic University of Health and Allied Sciences (CUHAS) within four hours of collection for laboratory analysis.

#### 4.5.2. Screening of MDR Bacteria

Stool/rectal swab samples were directly inoculated on MacConkey agar (MCA; HiMedia, Mumbai, India) supplemented with cefotaxime 2 μg/mL (MCA-C) for screening of extended spectrum β-lactamase producing Gram-negative bacteria (ESBL-GNB). Nasopharyngeal swabs were directly inoculated on 5% sheep blood agar (BA; HiMedia, Mumbai, India) for isolation of *S. aureus* while oral swabs were directly inoculated on BA and MCA plates for the isolation of bacteria pathogens. Plates were incubated aerobically at 37 °C for 18–24 h. Gram negative bacteria (GNB) isolated from oral swabs were further sub-cultured on MCA-C for screening of potential ESBL-PE. In-house prepared biochemical identification tests (Triple sugar Iron test [TSI] for sugar fermentation, CO_2_ and H_2_S production; Sulphur, Indole and Motility [SIM] for H_2_S and indole production and motility; Simmons citrate agar for utilization of citrate as the sole source of carbon; oxidase test for production of oxidase enzyme; and Christensen’s urea agar for urease enzyme production) were used for identification of GNB. For identification and confirmation of *S. aureus* (golden yellowish with β-hemolysis on BA plates), catalase, coagulase tests and DNAse agar were used. All procedures were performed as per standard operating procedures (SOPs) as previously documented [37].

#### 4.5.3. Antibiotics Susceptibility Testing

The disc diffusion technique, as documented by Kirby-Bauer, was used for antibiotics susceptibility testing (AST). Bacteria were suspended in sterile saline and turbidity were adjusted to 0.5 McFarland standard, then swabbed on Mueller Hinton agar (HI Media, New Delhi, India) plates. Antibiotics discs of ciprofloxacin (CIP 5 μg), gentamicin (CN 10 μg), tetracycline (TE 30 μg) and trimethoprim/sulphamethoxazole (STX 1.25/23.75 μg) (HiMedia, New Delhi, India) were tested for Gram negative bacteria from oral swab and stool/rectal swab samples. For Gram positive bacteria erythromycin (E 15 μg), tetracycline (TE 30 μg), gentamicin (CN 10 μg), ciprofloxacin (CIP 5 μg), clindamycin (CD 2 μg) and cefoxitin (FOX 30 μg) (HiMedia, New Delhi, India) were tested. Inoculated plates of MHA were incubated aerobically at 37 °C for 18–24 h. Interpretation of zones of inhibition measured in millimeters were performed as recommended by Clinical and Laboratory Standards Institute (CLSI, 2020) guidelines [38].

#### 4.5.4. ESBL Confirmation

Isolates were confirmed to be ESBL producers using disc approximation methods as previously described [39]. Two cephalosporins discs (ceftriaxone and ceftazidime) were seeded edge-by-edge with amoxicillin/clavulanic discs on MHA plates at a distance of 15 mm, inhibition of cephalosporins discs enhanced towards the disc of amoxicillin/clavulanic acid were interpreted as positive for ESBL production [40].

### 4.6. Data Management and Analysis

Data were collected using Epicollect5 (https://five.epicollect.net/), accessed on 10 August 2021 and analyzed using STATA version 15 software. The categorical variables were summarized as proportions while continuous variables were summarized as median [IQR; interquartile range] or mean (±SD; standard deviation) depending on the distribution. Chi square test was used to compare socio-demographic characteristics, ESBL-PE and MRSA colonization among HIV− and HIV+ children below five years of age. A stepwise variable selection algorithm was used was used to determine factors associated with ESBL- PE colonization. A *p* value of <0.05 was considered statistically significant. All analyses were conducted using STATA version 15.

## 5. Conclusions

Overall, the MDR carriage in children below five years of age was high and was increased by recent antibiotic use and a relative living in same household being admitted to hospital in the last three months. HIV+ children were more colonized by isolates resistant to uncommonly used antibiotics in the community. Antibiotic stewardship and guidance on appropriate use is extremely important in this setting to prevent resistant bacteria from further spreading in the community and healthcare facilities. There is a need to expand antibiotic stewardship programs, strengthening IPC practices to prevent the emergence and further spread of MDR pathogens in LMIC. Further studies are needed to establish the genotypes and mobile genetic elements responsible for persistence and transmission of MDR pathogens among children in the city of Mwanza, Tanzania.

## Figures and Tables

**Table 1:** Socio-demographic and clinical characteristics of enrolled participants.

		HIV− (N = 255)		HIV+ (N = 144)		*p*-Value
Characteristics		Frequency (n)/ Median [IQR]	Percentages (%)	Frequency (n)/ Median [IQR]	Percentages (%)	
Median [IQR] age in months		14 (9–22)		34 (22–44)		<0.001 **
Gender	Male	139	44.5	73	49.3	0.463
	Female	116	45.5	71	50.7	
Residence	Urban	250	98.0	130	90.3	<0.001 **
	Rural	5	2	14	9.7	
Immunization schedule	Completed	203	79.6	139	96.5	<0.001 **
	Not Completed	52	20.4	5	3.5	
Source of water	Tap water	241	94.5	122	84.7	0.001 **
	Pond/lake water	14	5.5	22	15.3	
Type of toilet	Modern toilet	244	95.7	120	83.3	<0.001 **
	Pit latrine	11	4.3	24	16.7	
Family size	Median	4 (3–5)		5 (3.5–6)		0.068
Care taker leve of edication	Primary	168	65.9	111	77.1	0.019
	Secondary	87	34.1	33	22.9	
Type of house	Brick	226	88.6	108	75	<0.001 **
	Mud	29	11.4	36	25	
Animal keeping	Yes	55	21.6	30	20.8	0.863
	No	200	78.4	114	79.2	
Duration of breastfeeding	Median [IQR] months	6 (5–6)		6 (6–6)		0.002 **
Use of antimalaria in the last month	Yes	26	10.2	8	5.6	0.111
	No	229	89.2	136	94.4	
Relative hospital admission in same household past 3 months	Yes	7	2.7	5	3.5	0.683
	No	248	97.3	139	96.5	
Antibiotics use over the past one month	Yes	227	89.0	130	90.3	0.694
	No	28	11.0	14	9.7	
ART us	Yes	NA	NA	143	99.7	NA
	No	NA	NA	1	0.3	
MUAC	SAM	34	13.3	3	2.1	<0.001 **
	MAM	221	86.7	141	97.9	

Key: MUAC = Mid Upper Arm Circumference, SAM = Severe Acute Malnutrition, MAM = Moderate Acute Malnutrition, NA = not applicable, ** Significant *p* < 0.01.

**Table 2:** Isolated pathogens from different sources among 399 participants by HIV status.

Isolate	Oral (N = 27)		Nasal (27)		Rectal (N = 278)	
	**HIV−**	**HIV+**	**HIV−**	**HIV+**	**HIV−**	**HIV+**
*K. pneumoniae complex*	3	8		NA	12	17
*E. coli*	4	1		NA	136	41
*S. aureus*		NA	21	6	NA	
MRSA		NA	13	3	NA	
*S. pneumoniae*	2	2		NA	NA	
Others	3 *	4 *		NA	45 **	27 **
Total	12	15	21	6	193	85

NA = Not Applicable; MRSA = methicillin resistant *Staphylococcus aureus*. * Others Oral; *A. hydrophila* (n = 1), *Acinetobacter* spp. (n = 1), *C. freundii* (n = 1), *S. pyogenes* (n = 1), *Pantoea agglomerans* (n = 1), Unidentified Gram-negative rods (n = 2). ** Others rectal; *A. hydrophila* (n = 17), *Citrobacter* spp. (n = 15), *E. cloacae* complex (n = 15), *Enterobacter* spp. (n = 1), *Pantoea agglomerans* (n = 5), Unidentified Gram-negative rods (n = 19).

**Table 3:** Percentage resistance and ESBL phenotypes of Gram-negative bacteria isolated from oral and rectal swabs.

Antibiotic	ORAL SWAB N = 18 (%)	RECTAL SWAB N = 278 (%)	*p* Value
Amoxicillin/clavulanic acid	4 (22.2%)	135 (48.7%)	0.025 *
Ceftriaxone	3 (16.7%)	132 (47.5%)	0.009 *
Sulfamethoxazole/Trimethoprim	15 (83.3%)	233 (83.8%)	0.583 **
Tetracycline	10 (55.6%)	186 (66.9%)	0.230 **
Gentamicin	2 (11.1%)	40 (14.4%)	0.516 **
Ciprofloxacin	3 (16.7%)	135 (48.6%)	0.007 *
Ceftazidime	2 (11.1%)	103 (25.8%)	0.019 *
Meropenem	2 (11.1%)	20 (7.2%)	0.394 **
ESBL	2 (11.1%)	92 (33.1%)	0.039 *

* Significant, ** Not significant.

**Table 4:** The resistance patterns of GIT isolates in HIV and non-HIV infected children (N = 278).

Antibiotic Agents	HIV− (n = 193)	HIV+ (n = 85)	*p* Value	Overall Resistance
Sulfamethoxazole/Trimethoprim	158 (81.9%)	75 (88.2%)	0.184	233 (83.8%)
Tetracycline	125 (64.8%)	61 (71.8%)	0.253	186 (66.9%)
Ciprofloxacin	92 (47.7%)	43 (50.6%)	0.654	135 (48.6%)
Gentamicin	16 (8.3%)	24 (28.2%)	0.000 **	40 (14.4%)
Ceftriaxone	87 (45.1%)	45 (52.9%)	0.226	132 (47.5%)
Amoxicillin/clavulanic acid	82 (42.5%)	53 (62.4%)	0.002 **	135 (48.6%)
Ceftazidime	66 (34.2%)	37 (43.5%)	0.138	103 (37.1%)
Meropenem	6 (3.1%)	14 (16.5%)	0.000 **	20 (7.2%)
ESBL	59 (30.6%)	36 (42.4%)	0.056	95 (34.2%)

** Significant *p* < 0.01.

**Table 5:** Factors associated with ESBL rectal colonization (N = 399).

Variable (N)	ESBL Colonization (n, %)	Univariate OR (95%CI)	*p* Value	Multivariable OR (95%CI)	*p* Value
**A: Child factors**					
Age (months)	* 19, IQR (10–29)	0.98 (0.97, 1.00)	0.154	0.98 (0.96, 1.00)	0.091
Sex					
Male (212)	56 (26.4)	1			
Female (187)	39 (20.9)	1.36 (0.85, 2.17)	0.194		
HIV status					
Negative (255)	59(23.1)	1			
Positive (144)	36(25.0)	1.11 (0.69, 1.78)	0.675	1.43 (0.81–2.52)	0.212
Residence					
Urban (380)	89(23.4)	1			
Rural (19)	6(31.6)	1.51 (0.56, 4.09)	0.418		
Antibiotics usage last month					
No (357)	79 (22.1)	1			
Yes (42)	16 (38.1)	3.45 (1.39, 8.58)	0.007	2.62 (1.1, 6.9)	0.04
B: Family factors					
Caretaker Education level					
Secondary (120)	30 (25.0)	1			
Primary (279)	65 (23.3)	0.91 (0.55, 1.50)	0.714		
Relative Admitted					
No (387)	88 (22.7)	1			
Yes (12)	7 (58.3)	4.8 (1.47, 15.36)	0.009	3.7 (1.1, 13.2)	0.03

* Median age of children colonized with ESBL-PE.

## Data Availability

Summary statistics have been included in this manuscript; raw data are available upon request from the Director of Research and Innovation of the Catholic University of Health and allied Sciences.

## Supplementary Materials

The following supporting information can be downloaded at: https://www.mdpi.com/article/10.3390/pathogens11030379/s1, Supplementary file S1: Multidrug resistance patterns among HIV− and HIV+ in children below five years of age.

## Abbreviations

AIDS	Acquired Immunodeficiency Syndrome
CD4	Cluster of Differentiation 4
CSF	Cerebrospinal Fluid
ESBL-PE	Extended –spectrum beta lactamase-producing Enterobacteriaceae
FBP	Full Blood Picture
HIC	High Income countries
HIV	Human immunodeficiency virus
IPC	Infection Prevention and Control
IQR	Interquartile Range
LMIC	Low- and Middle-Income Countries
MDR	Multidrug resistant
MRSA	Methicillin Resistant Staphylococcus aureus
MUAC	Mid-Upper Arm Circumference
NICU	Neonatal Intensive Care Unit
OI	Opportunistic infection
PICU	Pediatric Intensive Care Unit
PIC	Prevention Infection Control
SD	Standard Deviation
SIM	Sulphur, Indole Motility
TSI	Triple Sugar Iron Test
WBC	White Blood Cells
WHO	World Health Organization
