# Supplementary material for: High Fecal Carriage of Multidrug Resistant Bacteria in the Community among Children in Northwestern Tanzania
Source: Pathogens. 2022 Mar 21;11(3):379. doi: 10.3390/pathogens11030379 (PMC8955874; doi:10.3390/pathogens11030379)
Supplement: Supplementary file 1 [file pathogens-11-00379-s001.zip › pathogens-1535506-supplementary.pdf]

Supplementary file 1:

Multidrug resistance patterns among HIV and non-HIV infected children below five years of age.

MDR phenotype	Resistance Phenotype			HIV infected	Non-HIV infected	P Value	Overall
**MDR 1**	**SXT**	TET	CRO	49.4%	40.4%	0.163	43.2%
MDR2	SXT	TET	CIP	37.7%	31.6%	0.325	33.5
MDR3	SXT	TET	CN	22.4%	6.2%	0.000	11.2
MDR4	CRO	CIP	CN	16.5%	6.7%	0.012	9.7
MDR5	SXT	CIP	CRO	32.9%	26.4%	0.267	28.4
MDR6	TET	CN	CRO	17.7%	5.2%	0.001	7.0%
MDR7	TET	CIP	CRO	25.9%	19.7%	0.248	21.6%
MDR8	CN	TET	CIP	18.8%	5.2%	0.000	9.4%
MDR9	SXT	TET	CN	22.4%	6.2%	0.000	11.2%
MDR10	SXT	TET	CIP	22.1%	23.3%	0.780	22.9%
Overall MDR	-	-	-	58.8%	48.7%	0.120	51.8

Key: SXT Sulphamethoxazole/trimethoprim, TET = tetracycline, CIP = ciprofloxacin, CN=Gentamicin, CRO= Ceftriaxone.
